# Composite functional module inference: detecting cooperation between transcriptional regulation and protein interaction by mantel test

**DOI:** 10.1186/1752-0509-4-82

**Published:** 2010-06-10

**Authors:** Chao Wu, Fan Zhang, Xia Li, Shihua Zhang, Jiang Li, Fei Su, Kongning Li, Yuqing Yan

**Affiliations:** 1 Department of Bioinformatics and Bio-pharmaceutical Key Laboratory of Heilongjiang Province and State, Harbin Medical University, Harbin, Heilongjiang 150086, China; 2 Department of Genetics and Genome Biology, University of Toronto, Toronto, Ontario, Canada

## Abstract

**Background:**

Functional modules are basic units of cell function, and exploring them is important for understanding the organization, regulation and execution of cell processes. Functional modules in single biological networks (e.g., the protein-protein interaction network), have been the focus of recent studies. Functional modules in the integrated network are composite functional modules, which imply the complex relationships involving multiple biological interaction types, and detect them will help us understand the complexity of cell processes.

**Results:**

We aimed to detect composite functional modules containing co-transcriptional regulation interaction, and protein-protein interaction, in our pre-constructed integrated network of *Saccharomyces cerevisiae*. We computationally extracted 15 composite functional modules, and found structural consistency between co-transcriptional regulation interaction sub-network and protein-protein interaction sub-network that was well correlated with their functional hierarchy. This type of composite functional modules was compact in structure, and was found to participate in essential cell processes such as oxidative phosphorylation and RNA splicing.

**Conclusions:**

The structure of composite functional modules containing co-transcriptional regulation interaction, and protein-protein interaction reflected the cooperation of transcriptional regulation and protein function implementation, and was indicative of their important roles in essential cell functions. In addition, their structural and functional characteristics were closely related, and suggesting the complexity of the cell regulatory system.

## Background

Functional modules are basic units of cells that consist of molecules that work together to perform a desired biological function. The investigation of functional modules facilitates the understanding of the organization, regulation and execution of cell processes. Currently, several functional modules have been computationally extracted from the structural characteristics of biological networks, such as the transcriptional regulation networks, protein-protein interaction networks and metabolic networks [1-10]. However, these studies have mainly been performed on single networks, and cooperation between different types of networks is seldom considered.

The global cell network integrates single networks [11], such as the one governing transcriptional regulation, that appear to interact, rather than operate independently [12-16]. Recently, substantial cooperative structures called composite motifs have been discovered within integrated networks [13-15], and show functionally relatedness [13,15]. These composite motifs include two nodes, three nodes and four nodes motifs, such as composite pairs of co-transcriptional regulation and protein-protein interaction (CT-PPI). Three reports [13,15,17] showed that composite pairs of CT-PPI (C-pairs of CT-PPI) played important roles in cell function, especially in protein complexes which were also one kind of functional modules. But not all protein complexes are with high consistency between co-transcriptional regulation interactions (CTs) and protein-protein interactions (PPIs). Using yeast as model, Nicolas Simonis et al[18] and Kai Tan et al[19] discovered that protein complexes in the cell were not significant co-regulated.

Thus, we wished to investigate cooperation among different networks in a higher network structure hierarchy. In this work, we investigated the composite functional module of co-transcriptional regulation and protein-protein interaction (CT-PPI modules), and explored its structural and functional characteristics. Co-transcriptional regulation interactions (CTs) and protein-protein interactions (PPIs) are basic regulatory structures of transcriptional regulation and protein function. Our results showed that CTs and PPIs were highly consistent within the CT-PPI modules, indicating the important role of CT-PPI modules in cooperation between transcriptional regulation and implementation of protein function. We detected 15 CT-PPI modules that participated in essential cell processes including the oxidative phosphorylation pathway, RNA splicing, and DNA-dependent positive transcription regulation.

## Results and Discussion

**Figure 1:**
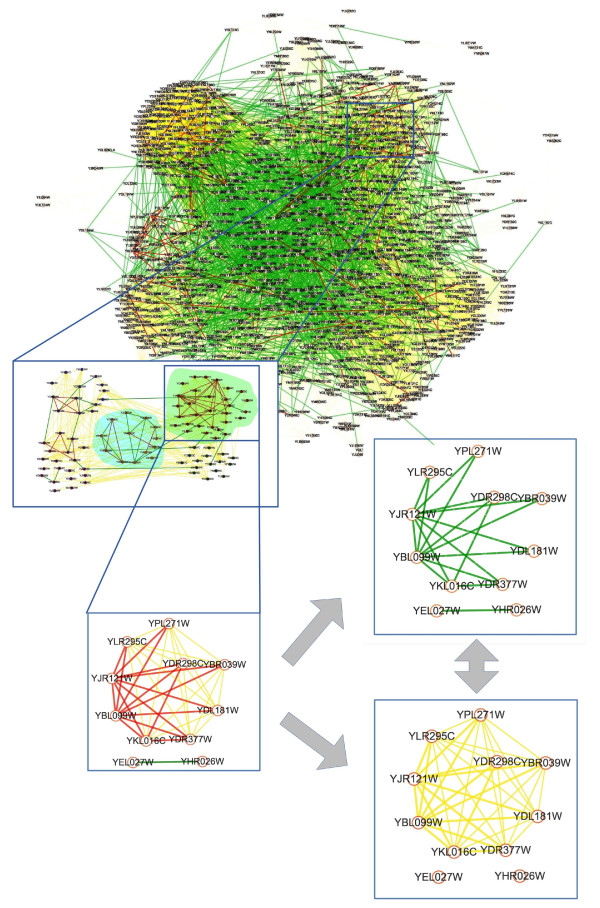
**The overview of the CT-PPI modules detecting method**. In the network representation, green lines between different nodes (proteins) represent the PPI pairs, yellow lines CT pairs, red lines C-pairs of CT-PPI. To be visually indicative, proteins in functional modules are highlighted in different shading groups. Note that the integrated sub-network of functional module would be separated into PPI and CT sub-networks to do the network comparison analysis.

We constructed an *S. cerevisiae *integrated network of 1107 nodes and 39,785 links (38,351 CTs and 1434 PPIs). In Figure 1, nodes represent genes, and coloured edges represent different types of links. Genes with the same GO annotation were regarded as a functional module, for a total of 300 functional modules in the integrated network which contained 100 cellular component (CC) term and 200 biological process (BP) term.

### Structural significance and functional coherence of composite CT-PPI pairs

**Figure 2:**
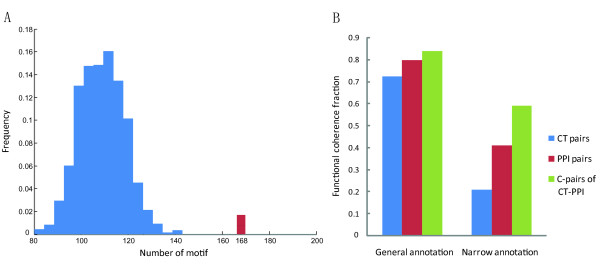
**Structural and functional characteristics of C-pairs of CT-PPI**. (A) The distribution of the number of C-pairs in randomized networks (blue bars) and in real network (red bar). (B) Functional coherence fraction comparison among C-pairs, CT pairs and PPI pairs. Using a background of GO annotation, the significance on CT-PPI/CT is *p *< 0.0001 and CT-PPI/PPI *p *< 0.0303. Using narrow annotation, the significance on CT-PPI/CT is *p *< 2.8384E-027 and CT-PPI/PPI *p *< 1.8466E-07.

Composite pairs of CT-PPI (C-pairs) are basic units that represent consistency between CTs and PPIs in the integrated network. And the presentation of C-pairs was different in our work and works reported before [13,15,17] (additional file 1 Figure S1A), for the integrated network was comprised by CT network and PPI network in our work but was comprised by PPI and transcriptional regulation interaction (TI) network in theirs'. We thought it could make the composite structure of C-pairs (e.g., CT-PPI modules) more concise (as Figure S1B showed) and help us detect CT-PPI modules with this presentation. C-pairs behaved as composite motifs in the integrated network because they occurred significantly more often than random (Figure 2A), this result also demonstrated the work reported before [13,15,17].

However, they also behaved as functionally coherent units. A C-pair was considered to be functionally coherent if both genes were annotated under the same GO term. Using a background of general (GO terms in our integrated network) or narrow (leaf terms) annotations, and considering only CC and BP branches, we compared the functional coherence fraction of 168 C-pairs to 38,351 CT pairs, and 1434 PPI pairs. A higher fraction of the C-pairs were functionally coherent, than the CT and PPI pairs (Figure 2B).

This result demonstrated that the cooperation between CTs and PPIs had important network structure and cell function effects. We searched for additional CT-PPI modules and investigated their characteristics.

### Detecting CT-PPI modules

Functional modules in single networks are usually detected from "structure to function", meaning that modules are searched first by network, then by functional annotation analysis [1-9]. We chose a "function to structure" method to detect CT-PPI modules by first defining the functional module, and then conducting topological analysis for consistency between CT and PPI sub-networks. The detail is shown in Figure 1. First, we constructed an integrated network of CTs and PPIs in *Saccharomyces cerevisiae*. Proteins were grouped into different functional modules according to their gene ontology (GO) annotations [20]. Finally, we used a network structure comparison Mantel test [21] to detect CT-PPI modules by their structural consistency between CT and PPI sub-networks in a given functional module.

We obtained 47 functional modules with a significant *r *value. We investigated the structural consistency of these functional modules to detect CT-PPI modules.

### Association between structural consistency and functional hierarchy of CT-PPI modules

**Figure 3:**
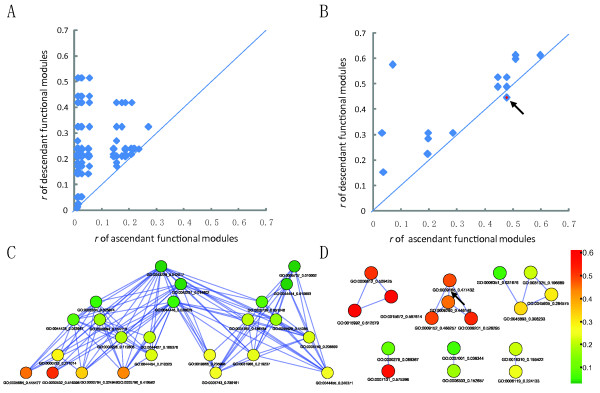
**The comparison between the statistic *r *values of ascendant/descendant functional modules**. (A) The comparison of *r *value pairs of ascendant/descendant functional modules in GO CC branch. (B) The comparison of *r *value pairs of ascendant/descendant functional modules in GO BP branch. Note that the *r *value pair (marked in black arrow) is GO:0009165-GO:0009260 where *r *value of the ascendant functional module is larger than that of the descendant one. (C) In the directed acyclic graph (DAG) of functional modules in GO CC branch, the colours of functional modules represent their corresponding parameter *r *value (D) In the DAG of functional modules in GO BP branch, different colours of functional modules represent their corresponding parameter *r *value. Note that the GO:0009165-GO:0009260 pair is marked in black arrow.

**Figure 4:**
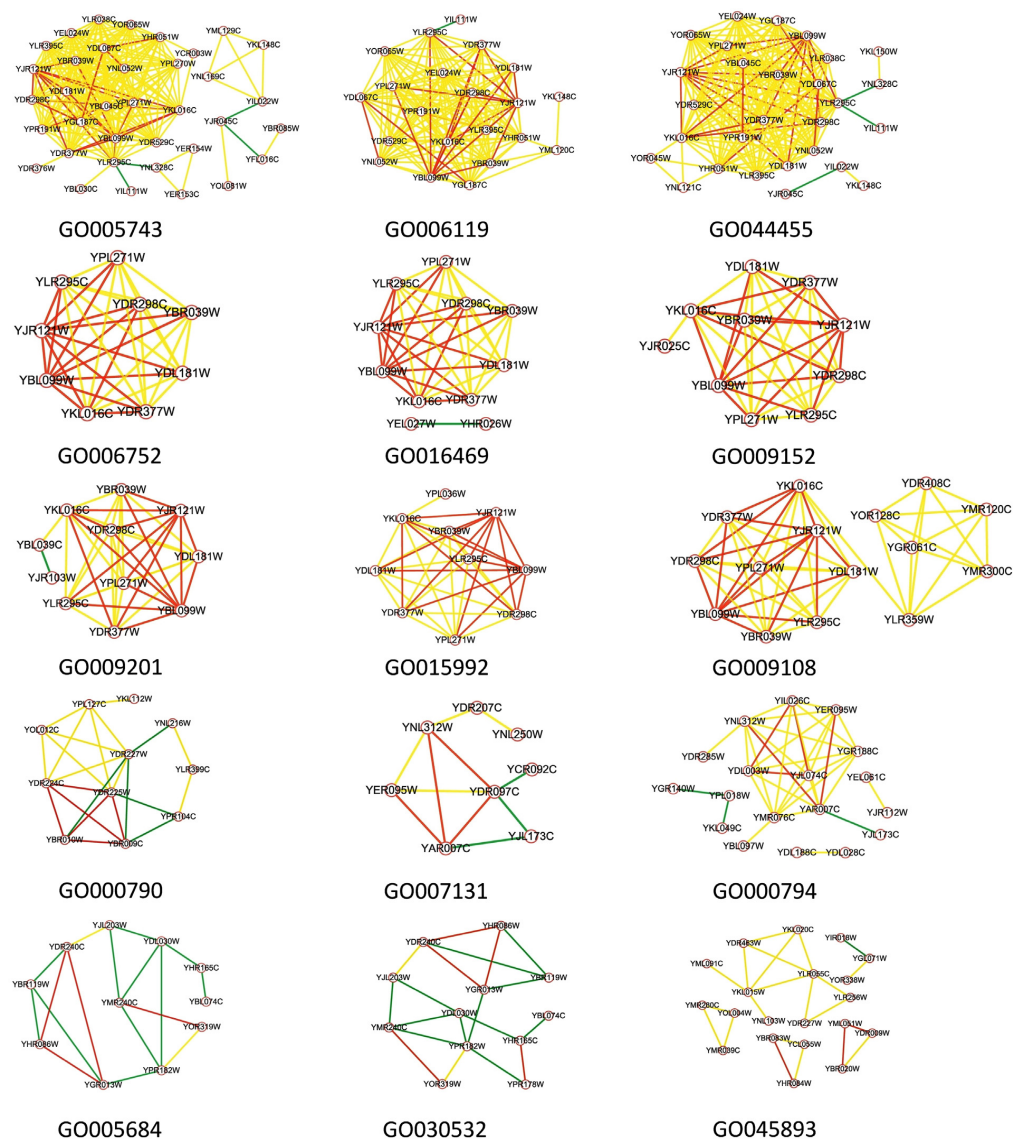
**Integrated sub-networks of 15 CT-PPI modules of CT-PPI**. In the network representation, green lines between different nodes (proteins) represent the PPI pairs, yellow lines CT pairs, red lines C-pairs of CT-PPI.

The structural consistency of CT-PPI modules was associated with their functional annotation hierarchy. We paired 41 of the 47 functional modules into 124 ascendant/descendant functional module pairs, with 6 excluded for lacking a relationship, according to their ascendant/descendant relationship in GO. Except for the GO:0009165-GO:0009260 pair, *r *values of the descendant function modules were greater than those of the ascendant modules (Figure 3). GO:0009165 and GO:0009260 shared the same number (17) of C-pairs, but the inconsistency of the other CTs and PPIs in GO:0009260 influenced the *r *value more than in GO:0009165. We used 0.2 as the threshold [22] for consistency between CT and PPI sub-networks of functional modules and obtained 25 functional modules. All *r *values were higher in the descendant, than in the ascendant functional modules. Choosing the descendent and isolated modules (functional modules with r > 0.2, but no ascendant/descendent relationships) yielded 15 CT-PPI modules (Table 1, Figure 4).

**Table 1:** CT-PPI modules

Type	GO ID	Description	R	Structure compactness
BP	GO:0015992	proton transport	0.6126	Y

BP	GO:0009108	coenzyme biosynthetic process	0.6085	Y

CC	GO:0016469	proton-transporting two-sector ATPase complex	0.5875	Y

BP	GO:0006752	group transfer coenzyme metabolic process	0.5702	Y

BP	GO:0009201	ribonucleoside triphosphate biosynthetic process	0.5263	Y

BP	GO:0009152	purine ribonucleotide biosynthetic process	0.4883	Y

CC	GO:0044455	mitochondrial membrane part	0.2404	Y

CC	GO:0005743	mitochondrial inner membrane	0.2392	Y

BP	GO:0006119	oxidative phosphorylation	0.2241	Y

BP	GO:0007131	meiotic recombination	0.5754	

CC	GO:0030532	small nuclear ribonucleoprotein complex	0.5164	

CC	GO:0005684	U2-dependent spliceosome	0.4455	

CC	GO:0000790	nuclear chromatin	0.4196	

CC	GO:0000794	condensed nuclear chromosome	0.3249	

BP	GO:0045893	positive regulation of transcription, DNA-dependent	0.3082	

### Global structural consistency of CT-PPI modules

C-pairs are the basic elements and locally consistent structures between CTs and PPIs in the integrated network. They also play important roles in the construction of CT-PPI modules, as CT-PPI modules were enriched with C-pairs (Table 2). In fact, CT-PPI modules were detected from the global consistency of CT and PPI sub-networks, rather than the local consistency (enrichment of C-pairs).

**Table 2:** Significance and corresponding rank of CT-PPI modules in enrichment analysis method

GO ID	C-pairs Number	P	Rank	GO ID	C-pairs Number	P	Rank
GO:0009201	17	8.3149E-48	1	GO:0005743	21	6.4295E-34	12

GO:0015992	17	3.0569E-46	2.5	GO:0000790	6	6.1192E-13	31

GO:0016469	17	3.0569E-46	2.5	GO:0030532	5	3.0351E-11	37

GO:0006752	17	7.5987E-45	4.5	GO:0007131	4	8.0637E-10	44

GO:0006119	20	5.6722E-44	6	GO:0005684	4	1.8409E-09	46

GO:0009152	17	1.9725E-42	7	GO:0000794	5	4.5027E-09	49

GO:0009108	17	2.277E-41	8	GO:0045893	3	0.00006537	93

GO:0044455	21	7.5852E-39	10				

Considering only local consistency generated many functional modules enriched with C-pairs. Of 198 functional modules containing C-pairs in the integrated network, 140 were enriched with C-pairs (*p *< 0.01). This relatively large number changed little as *p *decreased, so that even at *p *< 1 × 10^-10^, 41 functional modules were still found to be enriched with C-pairs (see Additional file 2), although this level of cooperation between CTs and PPIs associated with cell functions is implausible (Additional file 3). In addition, no clear relationship between *p *(representing the degree of enrichment of C-pairs in functional modules), and the functional hierarchy of such functional modules was found (Additional file 4).

### Structure compactness of CT-PPI modules

In single networks, links between genes in a module are more compact than links to genes in other modules [23]. If the inner link density *C_in_*, was greater than the outer link density *C_out, _*we considered the module compact (see Materials and Methods for detailed definitions of *C_in _*and *C_out_*). Compactness analysis of the functional modules separated the integrated network into CT and PPI networks, then examined the compactness of these sub-networks of functional modules. If both sub-networks were compact, we considered the integrated sub-network compact.

**Figure 5:**
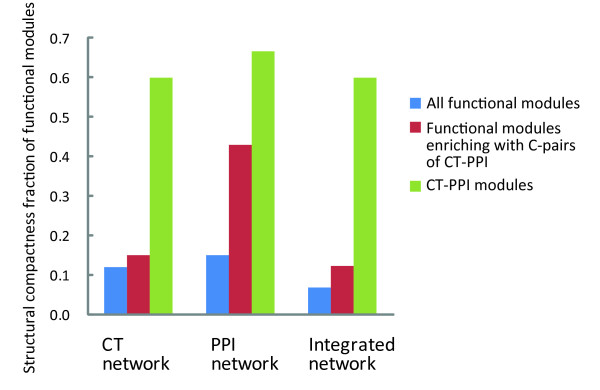
**The comparison of structure compactness fraction of CT-PPI modules with those of others **Columns (All functional modules, Functional modules enriching with C-pairs of CT-PPI and CT-PPI modules) represent structure compactness fraction in the three different kinds network forms (CT, PPI and integrated networks).

Compared with functional modules in the integrated network, and those enriched for C-pairs (*p *< 0.01), CT-PPI modules were more compact (Figure 5). Of 15 CT-PPI modules, 9 were compact in the integrated network (Table 1). This fraction (0.6) was much higher than the functional modules in the integrated network (0.07), and in those enriched for C-pairs (0.12). This showed that CT-PPI modules were not only modules in function, but showed modular behaviour in structure.

### CT-PPI modules involved in essential functions

The nine structurally compact CT-PPI modules were annotated for oxidative phosphorylation (Table 3), a critical metabolic pathway that produces adenosine triphosphate. The GO annotations of the 9 functional modules were closely related to the oxidative phosphorylation process. And the nine oxidative phosphorylation CT-PPI modules were closely related in structure, sharing 164/21 C-pairs, where the numerator is the total number of C-pairs, and the denominator is the number of unique C-pairs in the nine CT-PPI modules. These CT-PPI modules were combined and their 73 genes annotated by Kyoto Encyclopedia of Genes and Genomes (KEGG) [24], showing again that these genes were enriched in the oxidative phosphorylation pathway (*p *< 1 × 10^-32^). Furthermore, when we annotated the corresponding genes for the 21 unique C-pairs, 14 annotated in complexes III, IV or V of the oxidative phosphorylation pathway, while the corresponding genes number is 15. YDL181W lacked an annotation in the KEGG pathway system.

**Table 3:** Functions of CT-PPI modules

Functional description	GOID	TFs regulated C-pairs of CT-PPI
Oxidative phosphorylation	GO:0015992,GO:0009108,GO:0016469 GO:0006752,GO:0009201,GO:0009152 GO:0044455,GO:0005743,GO:0006119	HAP4(21)

RNA splicing	GO:0005684,GO:0030532	STE12(2) DIG1,PDR1,ABF1(1)

positive regulation of transcription, DNA-dependent	GO:0045893	GAL4(2) STE12,DIG1(1)

nuclear chromatin	GO:0000790	HIR2,HIR1(6) SWI4(1)
meiotic recombination	GO:0007131	MBP1(4)

condensed nuclear chromosome	GO:0000794	MBP1(5) SWI6(1)

*the number in () represent the C-pairs of CT-PPI regulated by the TF.

We investigated the transcriptional factors (TFs) regulating the C-pairs in the nine compact CT-PPI modules. Although many TFs regulated more than one gene in the nine CT-PPI modules, only *HAP4 *regulated C-pairs (Table 3). This suggested that *HAP4 *plays an important role in the regulation of the oxidative phosphorylation pathway, especially complexes III, IV and V, consistent with a previous report [19].

CT-PPI modules GO:0005684 and GO:0030532 appeared to affect RNA splicing, and shared TF genes *STE12 *and *DIG1 *with the CT-PPI modules GO:0045893 (Table 3). However, the term description of GO:0045893 in GO shows it plays roles in "the positive regulation of transcription, DNA-dependent". Transcription and RNA splicing are time-sequential, so shared TFs ensures the coordination of these two processes. We conclude that these CT-PPI modules participate in transcription, which, if interrupted, prevents successful production of mRNA and protein.

CT-PPI modules GO:0007131 and GO:0000794 both appeared to have roles in DNA structure changes in meiosis. CT-PPI modules GO:0000790 annotated as "nuclear chromatin", which is also involved in the chromosome formation. These three CT-PPI modules seemed to be involved in the maintenance and transmission of genetic material.

The above analysis shows that CT-PPI modules are involved in essential eukaryote cellular functions. Their network structure, evaluated as consistency of CT and PPI, reflects the cooperation of transcriptional regulation and implementing protein function, with this type of structure ensuring stable regulation. The network characteristic of CT-PPI modules ensures the stable regulation of their functions.

## Conclusions

Our results indicated that cooperation between CT and PPI is important to cell regulation. CT-PPI modules, which reflect the cooperation between CT and PPI in a module, were involved in essential cell functions. In addition, C-pairs, which reflected cooperation between CT and PPI motifs, were functionally coherent.

Our results also suggest that the structure and function of CT-PPI modules are closely related. Their network structure appeared to be conserved, as it coordinated two basic regulatory structures (CT and PPI). This type of structure could help ensure the stability of essential functions. Structural consistency and functional hierarchy in CT-PPI modules were associated, with their both functional and structural modularity. These findings reflect a close relationship between the structure and function of CT-PPI modules and show the complexity of cell regulation.

Many studies have investigated the relationship between the structure and function of special structures within networks, but findings have differed and the relationships have been ambiguous [13-15,25-31]. In eukaryotes, cell networks have undergone evolutionary pressure for billions of years, generating special structures. Molecular evolution hypothesizes that most evolutionary events behave non-directionally, so special structures that occur in the network do not always carry out corresponding functions. Therefore, we propose that investigating the biological meaning implied in the structures before exploring their functions is the most logical method of studying network structures.

## Methods

### Dataset

Experimentally identified interactions between TFs and their target genes in *S. cerevisiae *were extracted from chip-chip experiments [32], with data treated as Liao et al. [33] (*p *< 0.001). We obtained 4433 TIs for 113 TFs and 2400 target genes. If target genes shared TF (or TFs), we considered them co-regulated. In total, 167,708 CTs were found among 2376 genes.

Experimentally identified PPIs were extracted from the Database of Interacting Proteins (downloaded on September 2007) [34], yielding 17491 PPIs among 4392 genes, excluding homomultimeric proteins.

By overlapping the two data sets, we found 1856 genes with both types of links. For these genes, we selected GO items (layer > 5, annotated genes > 9 in BP and CC branches), and performed GO annotation analysis (downloaded on September 2007). Gene sets with ascendant and descendant GO terms were filtered for the descendant. We obtained 1107 genes annotated with 300 items (100 BP, 200 CC), with 38,351 CTs and 1434 PPIs. We defined a functional module as a gene group annotated in the same GO term in the integrated network.

### Structure significance analysis of C-pairs

We defined the number of C-pairs in the integrated network as *N_real_*, randomized the integrated network, and defined the number of C-pairs as *N_rand_*. The integrated network was randomized according to Yeger-Lotem et al. [13]. The integrated network was separated into CT and PPI networks. The two were randomized while keeping the degrees of nodes in each network unchanged, then integrated, for a total of 1000 randomizations. To our work, *N_real _*= 168, *N_rand _*= 109.8 ± 9.66 and the corresponding *Z_score _*= 6.03.

### Functional coherence analysis of C-pairs

Using the annotation information of the 1107 genes with 300 GO terms, we defined a gene pair (CT, PPI, C-pair) as a functionally coherent pair if both genes were annotated with at least one common term. Hypergeometric distribution was used to test whether C-pairs had a higher functional coherence proportion than either CT or PPI pairs, using the formula below:(1)

When comparing C-pairs with CT pairs,

*x *was the number of functionally coherent C-pairs in the integrated network,

*M *was the number of CT pairs,

*K *was the number of functionally coherent CT pairs, and

*N *was the number of C-pairs.

C-pairs were compared to PPI pairs in the same way.

Replacing the 300 GO terms with leaf GO terms and repeating the processing gave results under the narrow annotation system.

### Detecting CT-PPI modules

To detect CT-PPI modules, we used Mantel test, which accounts for distance correlations, to measure the consistency between the CT and PPI sub-networks of a functional module. The simple Mantel test was used to calculate the similarity of two symmetric matrices. Parameter *r *was a measure of similarity between matrices, and was the Pearson correlation coefficient of the corresponding elements in the lower or upper triangular parts of the two matrices. Parameter *p*, which measures the significance of the Pearson correlation coefficient *r*, was calculated as the probability that the number of *r *in the randomized networks would be equal to or greater than that in the real network.

The parameter *r *was calculated using the formula:(2)

Where *A *was the adjacency matrix representing the PPIs among 1107 genes. *A*_*ij *_was the Boolean value representing the interaction between protein *i *and *j*. When *A_ij _*= 1, PPI existed between *i *and *j*, and if *A_ij _*= 0 it did not. *B *was the adjacency matrix representing the CTs among 1107 genes. *B_ij _*was the boolean value representing co-regulation between proteins *i *and *j*. If *B_ij = _*1, CT existed between genes *i *and *j*, and if *B_ij _*= 0 it did not.

In this study, *r *represented the consistency between the CT and PPI sub-networks of a functional module, and *p *represented the significance of the consistency.

We used zt software [35], designed for Mantel tests, to calculate the consistency between CT and PPI sub-networks of the 300 functional modules. To test the significance of consistency, 10,000 randomizations were performed. We used *p *< 0.01 (FDR *q *value < 0.05) as the threshold.

### Enrichment analysis of C-pairs

We used a hypergeometric distribution to test the enrichment of C-pairs in a functional module, using the following formula:(3)

When analyzing the enrichment of C-pairs in a functional module *m*;

*a *was the number of C-pairs in the functional module *m*,

*X *was equal to , was the total number of genes in the integrated network,

*Y *was the number of C-pairs in the integrated network,

*Z *was equal to , and was the number of genes in the functional module *m*.

### Structure compactness analysis of CT-PPI modules

All the genes in a functional module were designated inner genes, and those outside a functional module were designated outer genes. For a functional module, the inner link density was defined as *C*_*in *_= *L *_*in *_/*G*_*in *_and the outer link density as *C*_*out *_= *L *_*out *_/*G*_*out *_[36].

*L_in _*was the number of links between inner genes,

*G_in _*was the number of inner genes with links to other inner genes,

*L_out _*was the number of links between *G_in _*inner genes and outer genes.

*G_out _*was the number of outer genes with links to *G_in _*inner genes,

If *C_in _*was greater than *C_out_*, we recognized the functional module as compact.

## Authors' contributions

CW, FZ and XL collected the data, carried out the detection and analysis of CT-PPI modules and wrote the paper. SZ and JL proposed and helped the analysis of C-Pairs. FS, KL and YY participated in the design and coordination of the study and helped draft the paper.

## Supplementary Material

Additional file 1 **Presentations of C-pairs and their composite structures in different integrated networks**. Additional file 1 is a figure showing presentations of C-pairs and their composite structure (e.g., CT-PPI modules) in our integrated network and in Yeger-Lotem E et al's and Haiyuan Yu et al's integrated network. In the figure, green lines between different circles (proteins) represent the PPI pairs, yellow lines CT pairs, red lines C-pairs of CT-PPI, blue dash direct lines from triangles (transcriptional factors) to circles represent TIs. (A) shows the presentations of C-pairs and their combinations (three nodes, four nodes) in Yeger-Lotem E et al's and Haiyuan Yu et al's integrated network (upside) and that in our integrated network (downside). (B) shows the integrated network of functional modules GO: 0000790 in Yeger-Lotem E et al's and Haiyuan Yu et al's integrated network (upside) and in our integrated network (downside). Click here for file

Additional file 2 **Functional modules enriching with C-pairs of CT-PPI with decreased *p***. Additional file 2 is a figure showing that the number of functional modules enriching with C-pairs of CT-PPI with *p *decreased. In the figure, Abscissa represents the significance of functional modules enriching with C-pairs of CT-PPI in -log(p) transform. Click here for file

Additional file 3 **Functional modules enriched with C-pairs of CT-PPI**. Additional file 3 is a table showing the functional modules enriching with C-pairs of CT-PPI. Click here for file

Additional file 4 **Statistic *p *comparison of the ascendant/descendant functional modules**. Additional file 4 is a figure showing the distribution of statistic *p *of the ascendant/descendant functional modules pairs enriching with C-pairs of CT-PPI. In the figure, (A) Distribution of the *p *of ascendant/descendant functional modules in CC branch of GO in -log(*p*) transform; (B) Distribution of the *p *of ascendant/descendant functional modules in BP branch of GO in -log(*p*) transform. Click here for file
