# Supplementary figures and images for: Composite functional module inference: detecting cooperation between transcriptional regulation and protein interaction by mantel test
Source: BMC Syst Biol. 2010 Jun 10;4:82. doi: 10.1186/1752-0509-4-82 (PMC2901225; doi:10.1186/1752-0509-4-82)

A

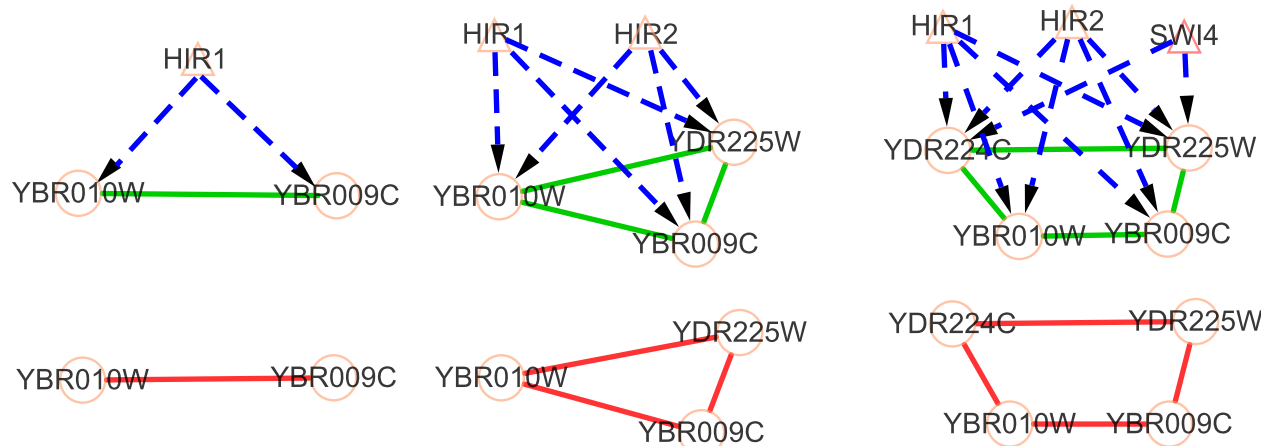

B

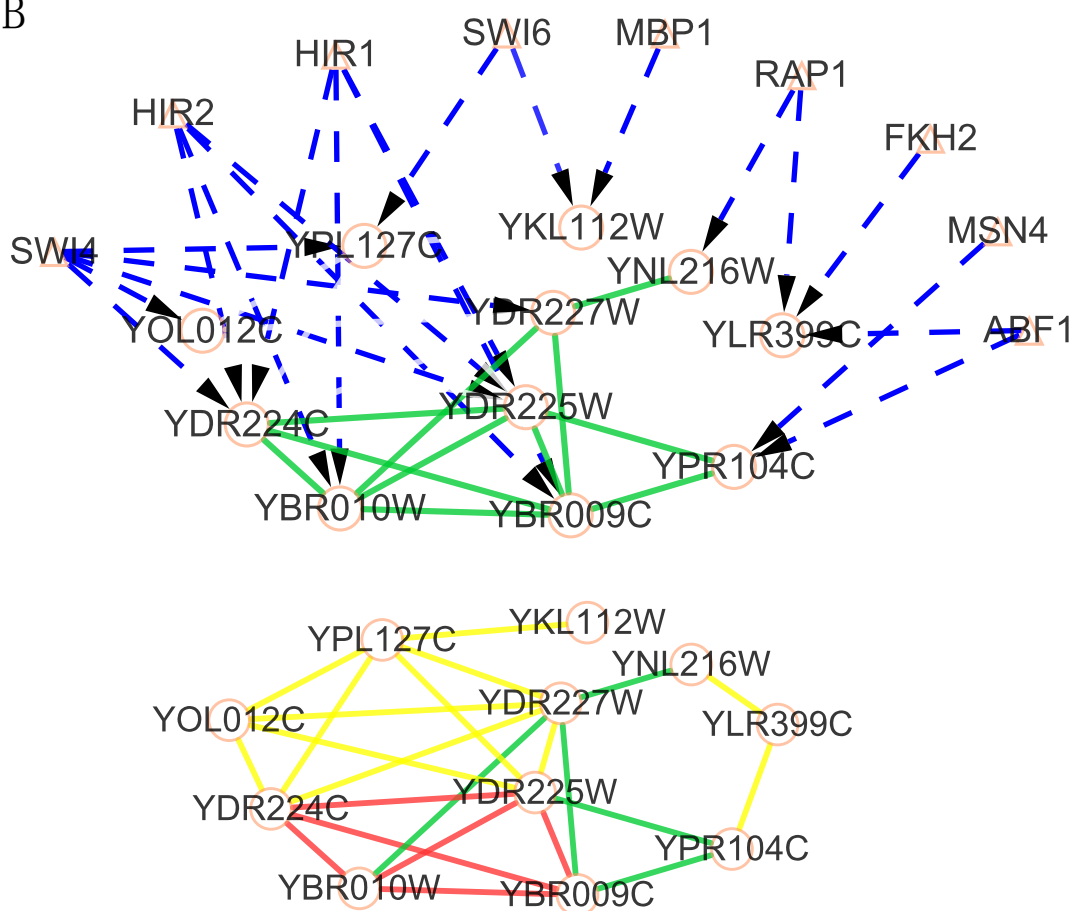

Supplement: Additional file 1 — Presentations of C-pairs and their composite structures in different integrated networks. Additional file 1 is a figure showing presentations of C-pairs and their composite structure (e.g., CT-PPI modules) in our integrated network and in Yeger-Lotem E et al's and Haiyuan Yu et al's integrated network. In the figure, green lines between different circles (proteins) represent the PPI pairs, yellow lines CT pairs, red lines C-pairs of CT-PPI, blue dash direct lines from triangles (transcriptional factors) to circles represent TIs. (A) shows the presentations of C-pairs and their combinations (three nodes, four nodes) in Yeger-Lotem E et al's and Haiyuan Yu et al's integrated network (upside) and that in our integrated network (downside). (B) shows the integrated network of functional modules GO: 0000790 in Yeger-Lotem E et al's and Haiyuan Yu et al's integrated network (upside) and in our integrated network (downside). [file 1752-0509-4-82-S1.PDF]

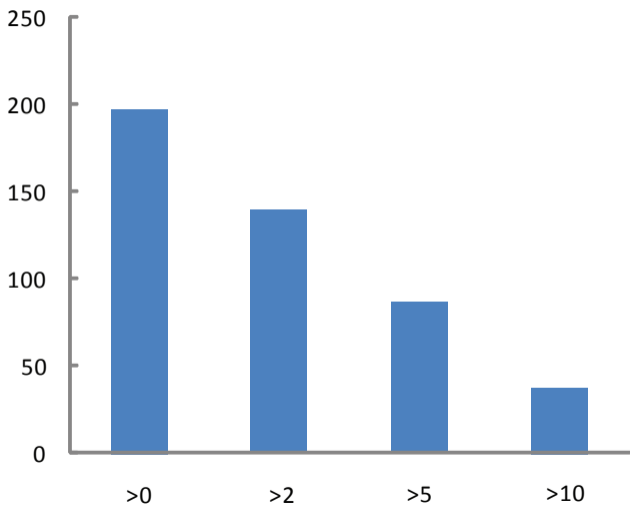

Supplement: Additional file 2 — Functional modules enriching with C-pairs of CT-PPI with decreased p. Additional file 2 is a figure showing that the number of functional modules enriching with C-pairs of CT-PPI with p decreased. In the figure, Abscissa represents the significance of functional modules enriching with C-pairs of CT-PPI in -log(p) transform. [file 1752-0509-4-82-S2.PDF]

A

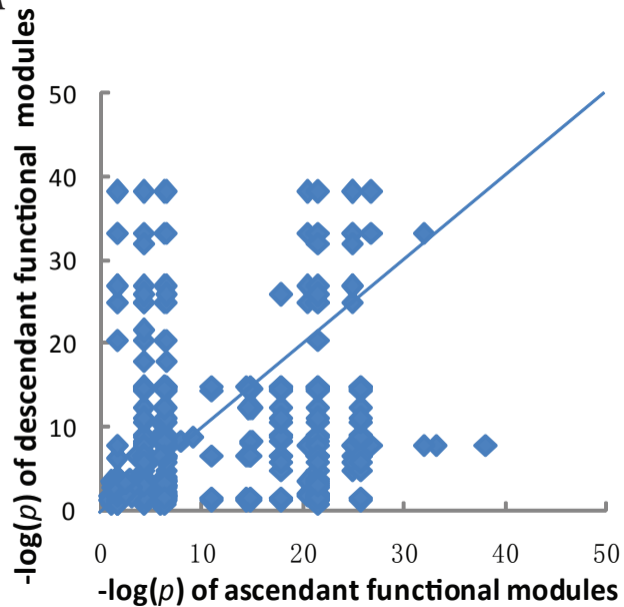

B

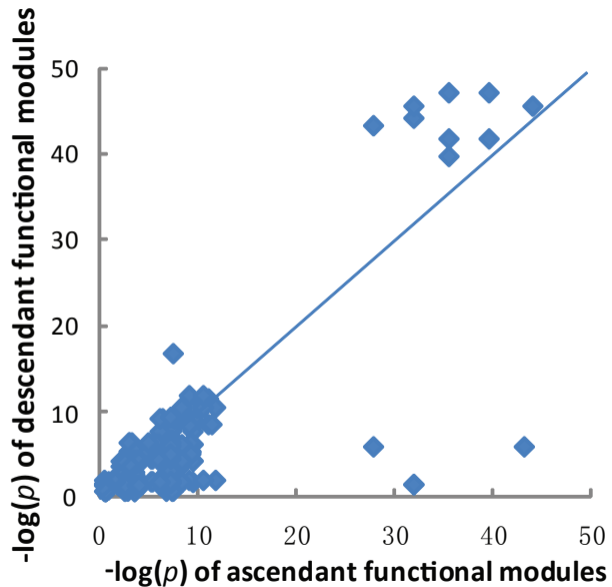

Supplement: Additional file 4 — Statistic p comparison of the ascendant/descendant functional modules. Additional file 4 is a figure showing the distribution of statistic p of the ascendant/descendant functional modules pairs enriching with C-pairs of CT-PPI. In the figure, (A) Distribution of the p of ascendant/descendant functional modules in CC branch of GO in -log(p) transform; (B) Distribution of the p of ascendant/descendant functional modules in BP branch of GO in -log(p) transform. [file 1752-0509-4-82-S4.PDF]
